# Experiences of clinicians engaged in report-back of individual chemical exposures in two pregnancy cohorts

**DOI:** 10.1186/s12940-026-01293-9

**Published:** 2026-04-02

**Authors:** Alina M. McIntyre, Katherine Franz, Cecilia Powderly, Nobel Hernández Otero, Katherine E. Boronow, Julia Green Brody, Phil Brown, Carmen Milagros Vélez Vega, Tamarra James-Todd, Marlee R. Quinn, Jennifer Liss Ohayon

**Affiliations:** 1 Silent Spring Institute, Northeastern University, 320 Nevada St #302, Newton, MA 02460 USA; 2 Department of Sociology and Anthropology, Department of Public Health and Health Sciences, Northeastern University, Boston, MA USA; 3 UPR Medical Sciences Campus, University of Puerto Rico Graduate School of Public Health, San Juan, Puerto Rico, USA; 4 Department of Environmental Health, Harvard T.H. Chan School of Public Health, Boston, MA USA

**Keywords:** Report-back, Environmental Health Knowledge, Clinicians, Endocrine Disrupting Chemicals

## Abstract

Reporting individualized chemical exposure results increases environmental health literacy, empowers study participants to reduce harmful exposures, and supports researchers in identifying and addressing exposure sources. Despite recommendations to increase clinicians’ roles in environmental health promotion, clinician-involvement in environmental health report-back remains limited. This study investigates clinician-facilitated environmental health report-back as a promising approach to results dissemination. We partnered with two pregnancy cohort studies to implement report-back of individual chemical results delivered by clinicians. Nine clinicians provided individualized exposure results to 87 participants across the two cohort studies. Following report-back sessions, we conducted semi-structured interviews to assess clinician experiences, perceived facilitators and barriers to report-back, and shifts in environmental health knowledge. Clinician-facilitated report-back was described as a rewarding process that enhanced clinicians’ ability to not only guide study participants through their individual chemical results but also expand their ability to provide environmental health information to other patients. Clinicians reported increased environmental health knowledge and adoption of personal exposure reduction habits. Clinicians from both cohorts discussed the challenge of communicating scientific uncertainty surrounding specific chemical exposures and related health outcomes. To address this, clinicians relied on structured training and materials developed by the study team to provide practical and actionable exposure reduction recommendations. Expanding training opportunities, embedding environmental health information into medical curricula and clinical practice, and developing guidance for clinician participation could advance communication of environmental exposure results.

**Supplementary Information:**

The online version contains supplementary material available at 10.1186/s12940-026-01293-9.

## Introduction

Reporting back individual-level chemical exposure results boosts environmental health literacy, defined as an ability for the general public to understand and act on the connection between environmental exposures and human health [1]. Report-back can also help researchers identify potential exposure sources by prompting discussions with participants about behaviors or environmental conditions that may contribute to elevated exposures and by drawing attention to results that warrant further investigation or targeted health protections [2–8]. Citing the benefits to researchers as well as to participants, the 2018 consensus report of the National Academies of Sciences, Engineering, and Medicine (NASEM) called for consideration of individualized chemical exposure results report-back in most studies [9]. Despite this, and unlike trends in genetics and clinical medicine [10–13], report-back is not yet a widespread practice in environmental health (EH) research. As the field continues to explore how to improve the reach and effectiveness of report-back, it is important to consider not only the content and design of exposure communications, but also the mechanism of delivery.

In previous studies, exposure results have been delivered by researchers, either through mail, digitally, or in-person meetings [7, 14, 15]. While researchers are knowledgeable of environmental health content, clinicians can play a critical role as health educators of patients [16]. Few studies have assessed clinicians’ involvement in the report-back of environmental health results.

Many environmental exposure studies take place in hospitals and other clinical settings, creating natural opportunities for the medical staff involved with research activities such as recruitment and study visits to also participate in returning chemical results to participants. Clinicians represent an understudied avenue for delivering report-back and shaping participants’ knowledge and engagement with environmental health. While research has shown that clinicians are trusted sources for health information [16], many are insufficiently prepared to discuss EH hazards and strategies for exposure reduction with patients [16, 17].

In a national online survey of over 2,000 obstetricians and gynecologists, less than 20% reported routinely asking about environmental exposures relevant to pregnancy outcomes, and approximately 5% reported any training on the topic [18]. Other studies surveying clinicians about EH discussion with patients report similarly low percentages [19–21]. Reported barriers to discussing EH exposures included general lack of knowledge, uncertainty about EH evidence, concerns about patient control over exposure reduction, and fear of causing patient worry [18]. Recent guidance from occupational and environmental medicine organizations, however, calls for clinicians to receive training in EH, as well as efforts to build clinician capacity for interpreting and communicating environmental exposures reduction [22–25].

While there is a significant body of research on the impact of individualized report-back, and best practices for designing reports, there is an absence of studies focused on integrating clinicians into this process. In collaboration with two large cohort studies, we examined the implementation of clinician-facilitated report-back of phthalate and phenol exposure results. Phthalates and phenols are synthetic chemicals widely used in consumer products including plastics, personal care products, and food packaging with the potential for widespread human exposure [26, 27]. These chemicals possess endocrine disrupting properties and are associated with adverse reproductive and developmental outcomes, making them of particular interest in studies of maternal and child health [28–31]. As a result, returning exposure results may be especially relevant for supporting participant understanding and engagement with exposure reduction recommendations.

We examined clinician experiences delivering environmental health report-back and assessed shifts in their EH knowledge and behaviors. We also aimed to identify practical strategies for involving them, as well as potential barriers to participation. We hypothesized that engaging clinicians in sharing chemical exposure results can offer EH educational benefits not only for study participants but also for the participating clinicians. By documenting clinician perspectives, our findings will inform future guidance for utilizing clinicians in report-back initiatives. Moreover, through our engagement with clinicians, we developed a model for training clinicians in returning personalized environmental exposure results.

## Methods

### Participating cohorts

To implement clinician-facilitated report-back, we partnered with the Puerto Rico Testsite for Exploring Contamination Threats (PROTECT) based at the University of Puerto Rico and Northeastern University [32], and the Environmental Reproductive and Glucose Outcomes Study (ERGO) based at the Harvard T.H. Chan School of Public Health [33]. PROTECT is a pregnancy and postpartum cohort receiving care at Puerto Rican clinics, and ERGO is a pregnancy and postpartum cohort based at Harvard teaching hospitals in Boston, Massachusetts. Both studies focus on the relationship between endocrine disrupting chemicals and pregnancy outcomes (i.e., metabolic health for mothers in ERGO, preterm birth in PROTECT) [29–31, 34]. All study protocols were reviewed and approved by the institutional review boards at Harvard University and the University of Puerto Rico.

### Report-back

Individual results reports were generated with Silent Spring Institute’s Digital Exposure Report-Back Interface (DERBI), which creates user-centered reports customized to a given study [35]. Results included exposure measurements for phthalates (both cohorts) and phenols (PROTECT only) in urine samples taken during pregnancy and/or postpartum. DERBI reports show individual results using text and figures that describe chemicals detected, related health concerns, exposure reduction strategies, and summaries of overall study results. DERBI reports were available in English and Spanish for PROTECT (see example report here: https://protect-en.reportback.org/r/report/demo) and English for ERGO.

Eligible participants had urinary measurements of phthalates and/or phenols and had not previously received individualized report-back. Participants meeting these criteria were randomly assigned to either the clinician-guided or self-guided report-back group. One ERGO participant was prospectively assigned to the clinician-guided group to ensure that all clinicians completed at least two report-back sessions. Participants assigned to the clinician-guided group (*n* = 72 PROTECT; *n* = 15 ERGO) received results during report-back sessions with trained clinicians, while participants in the self-guided group accessed their individualized DERBI reports independently through an email prompt. Participants were contacted via phone, email, or text message to schedule clinician-assisted report-back sessions. Participants in both groups received login information to access their DERBI reports after the report-back session or initial report release. 

### Clinician recruitment and training

We recruited and trained clinicians for each cohort to lead one-on-one conversations with cohort study participants. We defined clinicians as licensed healthcare professionals that have direct contact with patients, including for treating or preventing health issues, and are trained to communicate health information.

In collaboration with PROTECT, we recruited research nurses already affiliated with the PROTECT study. With the ERGO study, we recruited clinicians through the study team’s networks, including the Pediatric Environmental Health Specialty Unit (PEHSU) and Harvard’s teaching hospitals. We were unable to recruit clinicians affiliated with the parent ERGO study as planned due to clinician turnover at study-affiliated hospitals.

PROTECT and ERGO clinicians both attended training sessions to learn about chemical exposures, results communications, and the DERBI report that participants would receive. Both groups participated in mock report-back practice. PROTECT nurses attended in-person training sessions and were provided with fact sheets about the overall study and specific chemicals of interest and two instructional videos on the DERBI platform and returning results to participants. Trained PROTECT nurses then returned results to study participants in-person at a participating clinic, with discussions scheduled for 30 min. Before accessing DERBI, nurses provided a brief, individualized overview of study procedures, measured contaminants, and the platform’s features, including graphical displays. While we used this individualized approach, PROTECT has also historically conducted report-back within community-based settings that often include participants’ families [30, 32, 36]. ERGO clinicians attended training sessions over Zoom with similar content to PROTECT trainings. We also provided a report-back script for ERGO clinicians, as they were not previously affiliated with the study and requested additional structured support (see Supplemental Material). ERGO clinicians returned results to study participants virtually on a Zoom call that was scheduled for 30 min. For both cohorts, participants reviewed their personalized DERBI report for the first time during the clinician-facilitated report-back session and received an access code at the end of the session enabling them to log back into DERBI and review the reports on their own.

Training materials, including the report-back script provided to ERGO participants, were developed to provide specific language for clinicians to use for addressing topics that could be difficult to communicate, including scientific uncertainty around chemical exposures and associated health outcomes. For example, a part of this study’s report-back script reads:

*The chemicals in your ERGO report are found in almost everyone*,* and just because they were detected in your body doesn’t mean that you will get sick or that your pregnancy was affected. We are still learning about what levels of exposures affect health.*

In addition, training emphasized practical exposure reduction strategies and communication techniques to support participant empowerment. For example, the script provided to clinicians underscores the following:

*Most of these chemicals are broken down or leave the body quickly*,* often within a couple of days or hours. Because of this*,* simple actions can change your levels right away. I’ll let you look through some of these tips for reducing exposure – some involve choosing different products for personal care or cleaning*,* or making swaps to avoid plastics in the kitchen and throughout the home (including from food packaging).*

### Data collection and processing

We conducted semi-structured interviews over Zoom with clinicians after they had reported back results to all their assigned cohort participants. Clinicians received a $200 honorarium for returning results and a $50 gift card for interview participation. Questions in the interview guide centered around benefits and challenges associated with report-back, shifts in clinicians’ knowledge and concern about chemicals, whether report-back motivated them to provide EH information to patients outside of research studies, impacts on relationships with patients and researchers, and effective strategies for integrating report-back into clinical settings (see Supplemental Material). Interviews were recorded and transcribed by a third-party service and cross-checked for completion and accuracy by the study team.

### Analysis

Transcripts were coded in Dedoose, a qualitative analysis software program. Four researchers independently coded three initial transcripts using a codebook based on interview guide domains and questions (AMM, JLO, KF, CP). To assess intercoder reliability, researchers met to compare coded transcripts and share written memos (AMM, JLO, KF, CP). After reaching consensus on code definitions and application, the remaining transcripts were coded by a single researcher in the original language (AMM). We used thematic and content analysis methods to develop key concepts and themes [37] and calculated specific code frequencies. Quotes from PROTECT participants presented in this paper have been translated from Spanish to English (AMM, NHO).

## Results

### Study clinicians

Six clinicians reported back individualized results to 72 PROTECT study participants between July 2023 and July 2024, and three clinicians returned results to 15 ERGO participants between April 2024 and February 2025.

PROTECT clinicians were Puerto Rican nurses whose primary role in the research project was to assist with study recruitment at clinics and community health centers. Clinicians reporting back in ERGO included a primary care pediatric physician with a background in environmental health, a physician assistant with a background in obstetrics/gynecology and neurology, and a reproductive genetic counselor.

In interviews, clinicians reported gaining personal environmental health knowledge and adopting exposure-reduction behaviors themselves. Clinicians also described difficulties explaining scientific uncertainty and interpreting results for participants, given their own limited knowledge of the underlying science. Despite barriers such as cost of safer alternatives and lack of control over certain exposures, clinicians highlighted their unique role in producing practical, community-specific recommendations. PROTECT clinicians emphasized how their local knowledge allowed them to contextualize report-back results within participants’ lived environments. Finally, clinicians identified opportunities to improve report-back, including tailoring delivery format for specific study populations, integrating EH training into clinical practice, and ensuring support for adapting communication to participants’ varying levels of environmental health literacy. Four major themes were identified and are described with accompanying quotes from participants.

### Clinicians perceive report-back as both empowering and challenging

During report-back, clinicians described how both they and study participants learned new information about EH. Four PROTECT clinicians described feeling empowered by being able to offer information about personal exposures and solutions to study participants. One of these clinicians stated:

…a lot of learning [occurred] for both me and them [participants], and it was very gratifying to be able to share this information with the mothers and for them to understand, to learn ….

Almost all clinicians (*n* = 8) described impacts on their personal environmental health literacy from their study participation, including increases in environmental health knowledge and adopting personal exposure reduction actions, such as replacing plastic water bottles with stainless steel and replacing plastic food containers with glass. One PROTECT clinician shared her overall shift in perspective:

My thinking has changed a lot, my way of acting also changed…I think it has not only been a professional learning experience, but also a personal one.

At the same time, clinicians also navigated challenges related to communicating the scientific uncertainty with these chemicals and their related health effects, as well as explaining scientific results overall. Leveraging the report-back training, one ERGO clinician described how she provided actionable exposure reduction guidance rather than making definitive health predictions:

I didn’t want to be like, yeah, “this is going to be detrimental to your child’s health,” or “this is going to be detrimental to your own health in the future”…because I don’t think we can say that. I think it’s interesting, and it’s something we can look for, and it might increase your risk in the future… I gave the references that were in the material and said, “here are some steps that you can take to help reduce your [chemical] levels.”

A PROTECT clinician discussed how she relied on the practical exposure reduction recommendations to navigate situations where participants may not initially understand their chemical exposure results:

Well, my biggest concern was sitting down and explaining all of this to them, because there are mothers who can understand and others who may find it a bit complicated… it was like starting a conversation with them and speaking to them in their own language and telling them, look, this chemical is in this, you can find it, and I gave them many examples …to try to avoid it….

Other reported challenges faced during the report-back process included finding time to schedule participant report-back (*n* = 1), technology issues during report-back (*n* = 1), describing the significance of missing data (*n* = 1), and answering technical questions about specific chemical compounds (*n* = 1). Despite these challenges, all clinicians (*n* = 9) reported positive experiences with the report-back process and stated that they would be willing to participate in future report-back studies.

When asked why she would participate again as a report-back facilitator, a PROTECT nurse replied:

It’s really rewarding to be able to share that knowledge… For me, that’s what nursing is about: helping people take care of themselves and be well. And prevention education is prevention. I love the [report-back] project because it honors what we, our profession, nursing, are all about.

### Clinicians recognize the importance of community-specific exposures

Almost all (*n* = 8) clinicians noted that many of their patients may not be able to fully reduce their exposure to chemicals. For example, four out of nine clinicians mentioned the cost of safer products as a barrier for chemical exposure reduction among their patient population. Three clinicians also highlighted people’s limited control over their surrounding environment. As one PROTECT clinician described:

Sometimes it’s really difficult because I can control what I consume here at home, but the [outdoor] environment is something you’re exposed to and have no control over.

One ERGO clinician stated, “There’s a whole realm of things we either don’t know where they are right now or have no control over.”

Although clinicians identified barriers to exposure reduction among their patient population, they also emphasized their unique position to recognize and propose community-specific strategies. Among PROTECT clinicians, in particular, their lived experience in the community and regular interactions with participants through study activities positioned them to recognize broader exposure contexts and tailor exposure reduction recommendations. One PROTECT clinician, for example, described her experience with nail technicians:

In Puerto Rico, I work a lot with nail technicians…and we know there’s a high level of exposure. And sometimes I had women who tested high in those specific chemicals…and I would then stress to them the importance of taking care of themselves, of trying to wear masks…and other alternatives that could be implemented to try to take better care of their health.

While she would also explain to participants how dairy farms, a local industry, may be putting participants at risk, she indicated that she would emphasize actionable ways to reduce exposures despite community-wide factors: “… I’m not going to tell you to move [to a different house], but I can tell you that there’s a way to reduce your exposure…”.

In general, PROTECT clinicians’ familiarity with local occupational and environmental exposures enabled them to situate individual report-back results within the broader context of participants’ lives.

### Clinicians identify opportunities to expand and improve report-back

Reflecting on their own experiences, clinicians identified factors that can contribute to successful clinician-facilitated report-back. These included briefing clinicians with local or study population-specific exposure concerns, allowing clinicians to determine the best avenues for report delivery given their participant population (online, print-out, hybrid), and working towards the integration of EH training into existing clinical activities.

Clinicians’ participation in report-back highlighted an opportunity to expand EH education within broader clinical practice. Although clinicians reported limited bandwidth to perform report-back as part of their duties, half of the PROTECT clinicians engaged their larger patient population in discussions about EH and chemical exposure reduction after study participation. One PROTECT clinician described how participation in report-back expanded their general interest in environmental health:

I think [environmental health resources] should also be part of the orientation that the nurse or doctor gives to those women who are pregnant. In the same way as giving importance to prenatal pills, you have to take prenatal pills because those vitamins are important for the baby, give them the same importance….

Clinicians underscored how report-back needs to be adaptive to participants’ existing level of EH familiarity and interest. Moreover, there might be cases where clinicians need extra support from research staff in addressing detailed questions from study participants. As one ERGO clinician reflected:

I had people [participants] from all walks of life. I had people who didn’t really know anything about it and were just kind of interested. And then I had one woman [who] knew much more than I did about it. She was asking more in-depth questions. And I was like, I don’t know…. So, I kind of went through the full gamut of experience from the participants themselves.

As an ERGO clinician emphasized, genetic counselling is an advanced field when it comes to sharing results, and such established expertise can inform EH report-back. This clinician, a practicing genetic counselor, commented that the strategies used in genetic counseling and EH report-back are similar:

I think the overall structure and set-up of the [report-back] conversations were very similar [to genetic counseling]: you contact [participants], you provide education, you go through the reports, you answer questions, you walk through the visuals and what they mean to make sure that the participant or patient understands them. You’re giving a lot of patient advocacy, you’re giving resources. There’s that sense of empowerment and I’m on your side, and we’re going to figure this out together.

Interestingly, the ERGO genetic counselor commented on the difficulty in integrating EH professionals into the current clinical system:

It’s hard to get even genetic counselors in OB [obstetrics] offices. I think you’re going to have a really, really hard time getting an environmental counselor to be billable….

### The importance of training and effective report-back materials

When prompted, all clinicians reported that the training was adequate and described positive experiences. One PROTECT clinician described how: “Everything was very detailed and specific…” and another added: “I felt confident because I had good training”. An ERGO clinician commented that she particularly enjoyed the practice session.

When asked if there was anything that could be improved, one ERGO clinician commented on the desire for additional information in the trainings about the mechanism by which a particular chemical may lead to a disease outcome:

*To hear about*,* okay*,* we know this product exists. It’s a breakdown product of*,* you know*,* BPA…And studies about this tend to correlate with*,* you know*,* cognitive development or obesity or metabolic syndrome.*

Providing clinicians with a script ensured the sharing of practical exposure reduction strategies even when scientific uncertainties exist. Though few clinicians suggested improvements to the training, one suggestion highlighted the desire for additional scientific resources relating to chemical exposures such as key peer-reviewed articles about parabens and phenols.

**Fig. 1:**
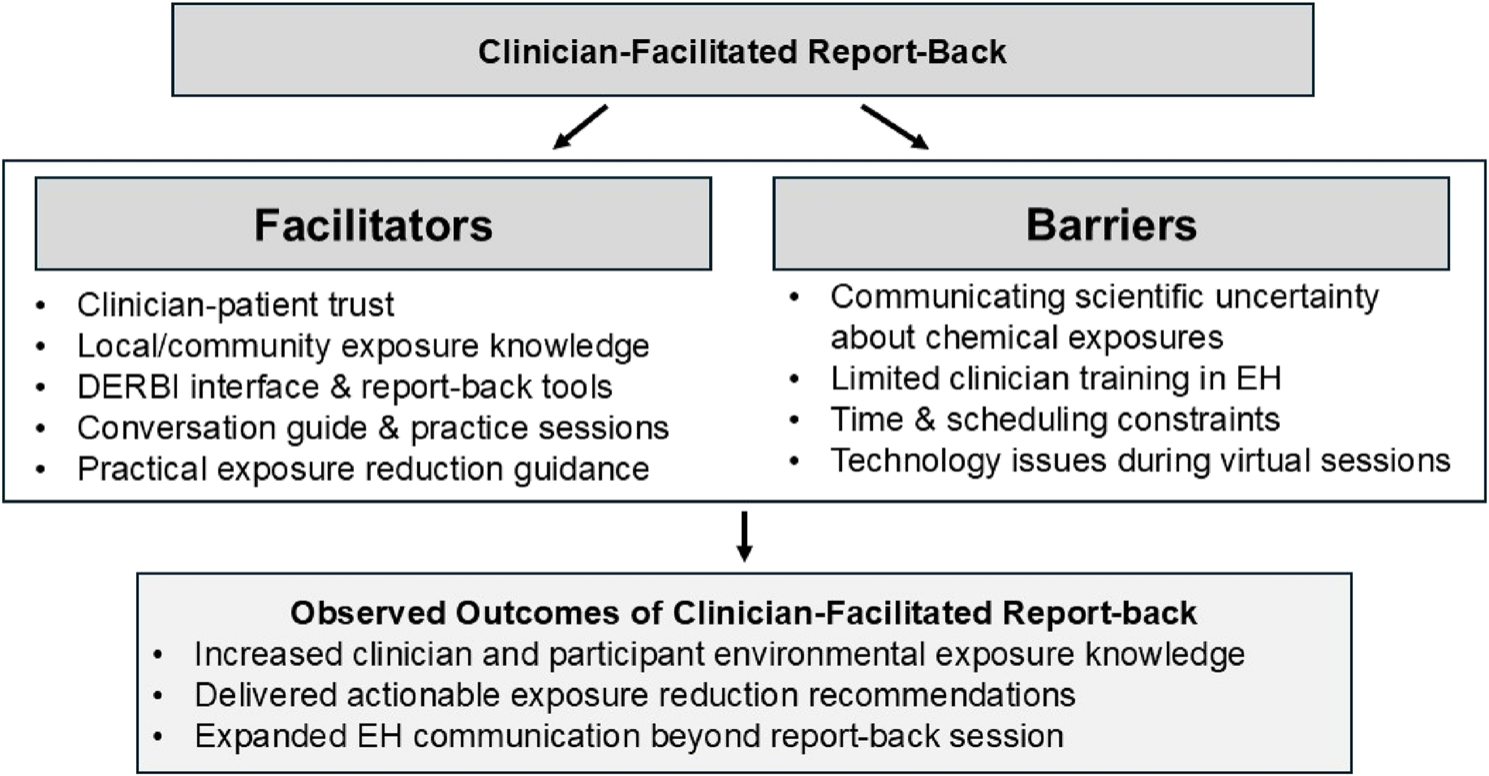
Conceptual framework illustrating facilitators, barriers, and observed outcomes of clinician-facilitated environmental health report-back identified through qualitative interviews with clinicians in the PROTECT and ERGO studies

These findings are summarized in a conceptual framework illustrating key facilitators, barriers, and observed outcomes of clinician-facilitated report-back (Fig. 1).

## Discussion

Clinicians described experiences with individual-level report-back as rewarding for both them and the study participants. Many described gaining new EH knowledge, adopting personal exposure reduction practices, and valuing their ability to give participants concrete, actionable guidance. Notably, about half of clinicians reported sharing environmental health information with patients outside of the study after their participation. At the same time, clinicians encountered challenges in translating chemical results with uncertain health implications, a reality inherent to the current state of chemical exposure research. This study focused on report-back of phthalate and phenol exposures; clinicians did not report substantial differences in communication strategies across specific chemicals. Some, however, noted that exposure reduction tips sometimes addressed broad chemical classes (e.g., phthalates as a class) and they felt unequipped to advise participants on why they might have a high result for a particular compound (e.g., is that compound related to a dietary source or household product?). While knowledge about specific exposure sources is often incomplete, future report design and training activities can bring in as much specific information as possible. Report-back approaches likely require adaptation for environmental exposures depending on the strength of evidence linking exposures to health outcomes and the availability of actionable exposure reduction guidance. To assist clinicians in communicating, training materials and scripts included language that addresses scientific uncertainty. In combination, these training experiences and tools illustrate how structured training and clear communication guidance can enable clinicians to convey exposure findings in actionable ways.

Clinician involvement in report-back was most successful when study clinicians were embedded in the research from the outset. PROTECT clinicians were present during all steps of the study protocol, from recruitment to sample collection and report-back, as hired staff. Additionally, their close familiarity with the patient population in Puerto Rico allowed them to discuss contextual factors impacting EH that were not in the reports. Aligned with the principles of community-based participatory research [38], PROTECT clinicians’ involvement in all stages of the research process and their closeness to the community enhanced the impact of the report-back. In this respect, integrating clinicians into EH report-back is likely more feasible and effective for research studies that have already established relationships with clinicians as part of the research protocol and sustain their engagement throughout the study. In contrast, clinicians recruited for report-back in ERGO were not affiliated with the ERGO study. Clinicians’ busy schedules and employee turnover at participating hospitals were major barriers to recruitment, ultimately limiting our sample size in ERGO. In cases where clinician-guided report-back is not feasible, online report-back or report-back facilitated by research staff also provides documented benefits for study participants [39–42]. In the PROTECT study, research staff have returned individual reports in community meetings where participants have had the opportunity to learn about overall study results, ask questions, interact with other participants, and pull researchers aside for individual conversations. Moreover, in addition to clinicians, other community-facing professionals such as social workers, housing support staff, or community health workers may be well positioned to support environmental health report-back efforts if appropriately trained. The communication strategies and training approaches described here are likely adaptable for research teams conducting report-back without formal clinical partnerships.

Our small sample size may limit generalizability to the broader clinician workforce in the United States or globally. Participating clinicians represented a range of sectors, and their perspectives were likely shaped by the context of their field. In addition, the report-back process relied on the DERBI platform and structured clinician training developed specifically for these studies, which may limit transferability to settings with different technological resources or training capacity. For example, DERBI’s digital reports aided ERGO clinicians in returning reports to participants via online video calls. Building report-back infrastructure into the design of new cohort studies from the outset may also improve feasibility by allowing research teams to establish clinician partnerships and training processes early in the study lifecycle. A key strength of the study includes the use of semi-structured interviews, allowing for in-depth understanding of clinician experiences with report-back to better inform future EH research and report-back efforts.

While previous studies have not tackled clinician-facilitated report-back of EH research results, other research has indicated challenges in integrating clinicians into research efforts more broadly. For example, a 2020 European-based study reported similar challenges such as lack of capacity among clinicians and study staff to learn extensive background information on EH study topics [43].

Researchers have also explored approaching study participants’ or patients’ environmental exposure experience and community (or place-based) context when considering their experience with illness or risk of becoming ill [2]. Our findings also support that contextualizing report-back within participants’ and patients’ lives can help bridge knowledge gaps and foster more meaningful clinician engagement. The source of report-back (for example, clinician versus researcher) may also shape participant experience, as differences in trust, communication style, or continuity of care could influence how individuals interpret and respond to environmental exposure information, an area that warrants further study.

### Opportunities for future clinician-facilitated report-back

For studies where clinicians are involved in research, affiliated clinicians can collectively learn from research staff and each other, building shared practices and knowledge that strengthens report-back as part of the research process. Existing initiatives such as the NIH Environmental Influences on Child Health Outcomes (ECHO) program and research studies embedded into hospital or clinical settings offer valuable opportunities to incorporate EH training into routine trainings or study designs. Relatedly, expertise from adjacent fields such as genetic counseling may offer transferable skills for report-back, as one clinician noted that her experience communicating genetic information aligned well with reporting back EH exposure information, both of which can be complex and may have uncertain interpretation. This underscores that including clinicians in research can have multiple benefits, including improving clinician EH knowledge as well as transferring the specialized training that clinicians receive in effectively communicating information to patients to EH report-back practices.

In addition, clinicians who are not involved in research may be interested in learning about EH and report-back on their own via published content, online offerings, or structured workshops. Many institutions and organizations actively conduct this work, for example, the Environmental Health Research Institute for Nurse and Clinician Scientists (EHRI-NCS) created a “train-the-trainer” EH mentorship program for clinicians and leading EH scientists, funded by the National Institute of Health (NIH) and National Institute of Environmental Health Sciences (NIEHS) [44]. There is also momentum to integrate EH content into medical and nursing school curricula [22, 24, 25]. However, workshops that train clinicians in the practice of EH report-back remain limited and could be developed as part of professional conferences, continuing education programs, and training opportunities.

General recommendations for involving clinicians in environmental report-back include implementing targeted clinician training on environmental exposures and health outcomes (as was developed for this project), continuing EH education and professional development activities, and building institutional support for clinician engagement in EH research collaborations [1]. Opportunities to advance the impact of report-back in clinical settings more generally include embedding “researcher-in-residence” programs that place research staff in clinical settings to support report-back efforts [45] and continuing to develop and adapt digital report-back tools for diverse patient populations [6, 35].

In this study, clinicians primarily framed their role in report-back as individual guidance and patient education; they did not describe engaging in policy advocacy or regulatory processes. Future report-back initiatives could therefore consider incorporating resources or pathways that connect exposure findings with broader structural or policy-level action. Beyond these initiatives, we recommend systemic solutions for enhancing clinical EH infrastructure in the U.S., such as establishing an adult version of the Pediatric Environmental Health Specialty Units [46] where clinicians could receive quick, free consultation, or an EH training program for clinicians that would be incentivized through debt repayment for public service. This type of model could build long-term capacity for clinician involvement in EH research and report-back at the national scale.

### Conclusions

This study offers novel insights into the firsthand experience of clinician-facilitated EH report-back. Our findings show that report-back can be a deeply rewarding experience for clinicians through advancing their EH knowledge and communication skills, and providing actionable guidance to participants. Meaningful clinician engagement is enhanced by integration into research teams, contextual understanding of participant communities, and sustained institutional support. Expanding EH and report-back training opportunities, embedding EH education in medical curricula, and developing systemic models for clinician participation can strengthen the impact of EH research and promote more equitable, informed communication of environmental exposure results.

## Supplementary Information

Supplementary Material 1.

Supplementary Material 2.

## Data Availability

The datasets generated and/or analyzed during the current study are not publicly available due to Institutional Review Board restrictions related to participant confidentiality, as the data consist of qualitative interview transcripts. De-identified excerpts or summary data may be available from the corresponding author on reasonable request.

## Abbreviations

DERBI: Digital Exposure Report-Back Interface
ECHO: Environmental Influences on Child Health Outcomes
EH: Environmental health
EHRI-NCS: Environmental Health Research Institute of the National Children’s Study
ERGO: Environmental Reproductive and Glucose Outcomes Study
NASEM: National Academies of Sciences, Engineering, and Medicine
NIEHS: National Institute of Environmental Health Sciences
NIH: National Institutes of Health
PROTECT: Puerto Rico Testsite for Exploring Contamination Threats
