# Supplementary material for: Experiences of clinicians engaged in report-back of individual chemical exposures in two pregnancy cohorts
Source: Environ Health. 2026 Apr 2;25:45. doi: 10.1186/s12940-026-01293-9 (PMC13169688; doi:10.1186/s12940-026-01293-9)
Supplement: Supplementary file 1 — Supplementary Material 1. [file 12940_2026_1293_MOESM1_ESM.pdf]

Report-Back of ERGO Research Results with DERBI: Clinician Checklist

➔ Before the Zoom call begins, prepare by finding the participant's access code (in your calendar invite for the call) and navigating to www.reportback.org/ergo in a browser window.

To do	Sample Script	Done?
**Thank participant**	*Welcome! Thank you for setting up this time to go over your results from the ERGO study. My name is [_]. What would you like me to call you?*	
**Explain purpose of session**	*We'll be looking at your personal report together.*	
**Explain future access to report**	*At the end of this call, I'll give you a passcode to access your DERBI report on your computer or phone. You can come back to it at any time to see your results and other information, and I'll show you how to save or print it too.*	
**Share screen with DERBI report**	*I'll go ahead and share my screen -- please feel free to stop me any time to ask questions!*	

➔ Prior to logging in, provide a quick overview of the study and the contents of a DERBI report. Then, click "Start Here" and enter participant's access code on the login page.

To do	Sample Script	Done?
**Verify that participant can view the report and read text**	*Can you read the text, or should I zoom in a bit?*	
**Provide study overview**	*It's been a while since you participated in the study, so as a reminder,*<br>*the goal of the ERGO study is to learn more about how chemicals that people come in contact with every day affect health during and after pregnancy.*<br><br>*You might remember giving urine samples at your regular visits during pregnancy and postpartum. ERGO tested those samples for chemicals known as phthalates, which we'll talk more about in a couple of minutes.*	
**Provide report overview**	*This report will show the levels of phthalates found in your samples, how they compared with other people, and what researchers have learned so far about how phthalates can affect health. It also tells you more about how you can reduce your exposure.*	
**Log into report with report access code**	*Again, you'll have access to see your report on your own later. For now, I'll log in for you.*	

➔ After logging in, read the participant's headline. Then, scroll down to the heading of "Health Concerns." Individual results and tips for reducing exposure will be discussed in more depth on the phthalates page.

To do	Sample Script	Done?
**Read participant's headline about phthalates**	*OK, now we're on the summary page where each participant sees a headline pointing out a finding from their results. For you, the headline says:*<br><br>*[**Example headline**:] During pregnancy, your sample had levels of phthalates that were sometimes higher and sometimes lower compared to other reproductive-age women in the U.S. At your postpartum visit, your sample had higher levels of a phthalate than 75% of reproductive-aged women in the U.S.* ***[will vary by participant]***<br><br>*Did you have any questions about your headline?*	

	We'll take a look at those levels in a couple of minutes. First, I want to talk a little more about phthalates and health.	
**Discuss endocrine disruptors and links to glucose intolerance**	*If I scroll down, you can see information about chemicals and health. During pregnancy, hormones sometimes go up to levels that interfere with the ability of insulin to regulate blood sugar, leading to problems like gestational diabetes. The ERGO Study is designed to learn whether endocrine disruptors like phthalates increase the chance of these types of issues. Endocrine disruptors can also affect fertility, child development, the nervous system, and cancer. Small amounts of endocrine disruptors can affect health. In case you aren't familiar with the terms, endocrine disruptors interfere with the body's hormones. Hormones travel in the blood to control activities in the body.*	
**Discuss scientific uncertainty and relationships to health**	*The chemicals in your ERGO report are found in almost everyone, and just because they were detected in your body doesn't mean that you will get sick or that your pregnancy was affected. We are still learning about what levels of exposures affect health.*	
**Pause for questions**	*Any questions so far?*	
➔ Provide background about phthalates as a chemical group before slowly scrolling through the results graphs.		
**Describe sources**	*OK, I'm going to click on your headline to see more details. Although there are many different phthalate chemicals, they share some similarities and are often found in the same types of products. Some phthalates make plastics more flexible and durable. These phthalates are added to products like vinyl flooring, shower curtains, toys, and food packaging.*<br><br>*Other phthalates help products hold their fragrance and color, so they are added to personal care products like shampoos, perfume, air fresheners, and printing inks.*	
**Describe health concerns**	*As I mentioned, many phthalates can harm the development of the reproductive system in babies and affect fertility, and some are suspected to increase the risk of cancer. Some phthalates contribute to allergies and asthma, and studies have also shown associations between phthalates and diabetes and cardiovascular disease risk.*	
**Show tips for reducing exposure, and ask about consideration**	*Most of these chemicals are broken down or leave the body quickly, often within a couple of days or hours. Because of this, simple actions can change your levels right away. I'll let you look through some of these tips for reducing exposure – some involve choosing different products for personal care or cleaning, or making swaps to avoid plastics in the kitchen and throughout the home (including from food packaging).*<br><br>*Are there any tips in particular you'd consider trying?*	
**Explain how to read the graphs, using first graph as an example**	*As we scroll down the page you can see your levels for each of the phthalates. The orange dots show your personal results for each sample that you provided for testing. The graphs show how you compare with other people. The blue dots show other peoples' results during pregnancy, and the purple dots show results at postpartum. The gray dots are samples where the chemical wasn't found. The black arrow at the bottom shows the middle value for the participants in ERGO, while the American flag represents the typical value for women in the U.S.*	

	*[**Example result]**: In this graph, your level at your first visit during pregnancy was higher than both the typical U.S. and ERGO measurements, but your second visit was lower. Your visit at postpartum was close to the middle.* ***[will vary by participant]***	
**Pause for questions**	*Do you have any questions about the graphs?*	
**Scroll briefly through each graph, stopping to describe notable results**	*[**Example result**:] I'm seeing a couple chemicals that were higher at a single visit - MECPTP at postpartum, and MCOP during pregnancy. Near the bottom of the page, there are a few phthalates that were never detected in your urine. If you want to see the exact measurements for each of your results, we can click on the orange dot.* ***[will vary by participant]***	
**Acknowledge any feelings of surprise, worry, or curiosity**	*Sometimes people are surprised that chemicals linked to health problems are so common in people.* ***One important reason for this is that in the U.S. companies don't have to test chemicals for effects on health before they put them into products. They can also use chemicals that are similar to those that have already been shown to be harmful.***<br><br>*Now that we've looked at your individual results, let's look at some of the overall findings from the study. So, I'm going back to that summary page that we saw before.*	
➔ Allow participant to read through the summary sentences of the different findings, then choose one or two to review more closely.		
**Show the Table of Contents for overall study results**	*Researchers have learned a lot from everyone who participated in ERGO. They found that most of the phthalates were commonly found in people, and using certain products increased their levels. Another finding was that people who used hair oils delivered earlier. This list shows just some of the study findings.*<br><br>*I'll let you read through these and pick one or two to look at together.*	
**Read one or more findings together, selecting based on participant interest if possible**	*[**Example overall study results:]** Use of hair oils during late pregnancy was associated with earlier delivery.*<br><br>*Among ERGO participants, about 7% delivered their baby preterm (before 37 weeks of pregnancy). Non-Hispanic Black participants gave birth about one week earlier on average than non-Hispanic White participants, which mirrors national trends. Stressors like discrimination likely play a role, and chemical exposures could be important too.*<br><br>*When researchers looked specifically at hair products, they found that non-Hispanic Black participants were more likely to report frequent use of products such as hair oils, hair lotions, and leave-in conditioners. Participants who used hair oils daily during late pregnancy gave birth 8.3 days earlier on average than those who did not use hair oils.* ***[will vary by participant]***	
**Pause for questions or review of any results**	*There's a lot of information on this page. Before you go, I just want to walk through the other pages – you can look at these in more depth on your own.*	
➔ Briefly introduce the "About the Study" page		

**Describe "About the Study" page**	*The "About the Study" page gives some more background on the ERGO study.*	
➔ Briefly introduce the "Community Action" page		
**Describe "Community Action" page**	*The "Community Action" page gives ideas on how to make a difference in your community, such as through voting or joining an environmental group, like the Alliance for a Healthy Tomorrow or Massachusetts Sierra Club. Even small steps like telling a friend or family member about what you've learned can make a difference for reducing harmful exposures.*	
➔ Explain how participants can print or save a pdf version of the report		
**Explain how participants can print or save a pdf version of the report**	*The last thing I want to show you is where to print or save a pdf of your report. On the left-side menu, click "Download Your Report." This will start a download of a pdf version of your report.*	
➔ Show participants where to log into their reports, and deliver access code		
**Confirm participant has means of recording URL and access code**	*Did you have any questions before I give you your access code? OK, now I'll share your access code so you can return to your report on your own. Do you have a pen and paper handy, or can you make a note on your phone?*	
**Inform participant of URL and access code**	*You'll want to visit www.reportback.org/ergo. I'm also going to put that url in the chat. There will be a blue button that says "start here" and that will bring you to a login page. Then, you'll enter an access code. Your code is XXXXX [**will vary by participant**]. Do you need to hear that again?*<br><br>*You'll also receive an email with this URL and access code so please check your SPAM folders if you don't see it. We didn't have a chance to go over all the information in your report, so I'd encourage you to log in on your own.*	
➔ Show participants where to log into their reports, and deliver access code		
**Direct to ERGO team for questions**	*If you have any questions, don't hesitate to reach out to the ERGO team. Their contact information is in report and I'm happy to provide it now over the phone as well if you'd like.*<br><br>*[**If contact information is requested**:] You can call them at [insert phone number], or send an email to [insert study email].*	
**Reminder about post-survey**	*One last thing. You may remember that you did a pre-survey before getting your results and ERGO will be contacting you soon about a post-survey. Your input will help us improve the reports for people in future studies.*	
**Thank participant**	*Thanks again for participating in ERGO and this added study on report-back!*	
