# Supplementary material for: Experiences of clinicians engaged in report-back of individual chemical exposures in two pregnancy cohorts
Source: Environ Health. 2026 Apr 2;25:45. doi: 10.1186/s12940-026-01293-9 (PMC13169688; doi:10.1186/s12940-026-01293-9)
Supplement: Supplementary file 2 — Supplementary Material 2. [file 12940_2026_1293_MOESM2_ESM.pdf]

Clinician Report-Back Interview

Questions for Health Professionals Returning Results

I. Background

1. Please briefly tell me about your training and work experience.

II. Report-Back Experiences and Implications of Report-Back for Environmental Health Knowledge, Efficacy, and Concerns

I'd like to ask you some questions now about your interactions with individuals receiving their exposure reports and about how participating in report-back influenced your thinking about environmental chemicals.

2. First, could you please tell me your impressions of the primary goals and overall findings of <*insert name of study, either ERGO or PROTECT*>?
    a. How did sharing results with study participants influence your understanding of the study? *Prompt:* Did participating in report-back help you understand the health outcomes and implications of the study? *If yes*, in what ways?

3. What was your experience like when sharing personal exposure reports with individual study participants?

4. Can you tell me some more about your conversations with individuals participating in the study?
    a. What information did you present to them?
    b. What questions did people ask about their results?
    c. Did you talk with them about the implications of chemical exposures for their health? *If yes*, what did you talk about? How did they react?
    d. Did you recommend any specific ways for them to reduce their exposures? *If yes*, what information did you provide? How did they react?

5. What was your preexisting level of concern about environmental chemicals prior to participating in report-back?
    a. How have your concerns about links between chemicals and health outcomes changed since sharing individual results with participants?

6. What ethical issues were you concerned about in this process? (*Prompts:* Were you concerned that participants would get overly anxious? That they would not have the ability to make changes to reduce personal exposures?)
    a. How did these concerns influence your interactions with your patients?
    b. Did you have any hesitations about sharing results with pregnant patients or those who recently gave birth or have young children? *If yes*, why? *If no*, why not?

7. What were participants' reactions to their results? How did you respond?

8. Are there certain types of environmental exposures for which you think clinicians should be more or less inclined to share results with their patients? Has report-back changed your opinions on what types of environmental exposures to share?

9. Are there particular exposure sources that you are most concerned about with respect to your patient population?
    a. Are there exposures that you would advise prospective parents, expecting people, or families with young children to avoid or reduce?
    b. Did reporting back results to participants shift your awareness or level of concern about these specific exposures?

10. What amount of control do you think patients have over their exposure to environmental health hazards?
    a. What ways, if any, can you help patients reduce their exposures? In what ways, if any, are you limited in helping patients reduce their exposures?
    b. Did sharing chemical results with participants change your ability to help your patients in general reduce their exposures? *If yes*, in what ways?

11. What worked for you when returning results to participants? What difficulties did you experience, if any, when returning chemical exposure results with people?

12. What advice do you have for other research teams and clinicians who would also share participant chemical results?

13. Could you describe whether the environmental health and report-back training that you received was adequate?
    a. What additional information or training could have prepared you further to share individual results? *Prompts:* Would it help to have longer or multiple training sessions, more interactions with study investigators, best-practice guidelines, or something else?

III. Implications of Report-Back for Clinical Care and Relationships to Patients and Study Team

Now I'd like to ask you about whether sharing results has changed how you provide clinical care, or your relationships to patients and researchers.

14. Did sharing results with study participants influence the ways in which you interact with patients outside of the study? *If yes,* please describe how this has shifted your interactions.
    a. *Prompt:* For patients outside of the study, have you provided environmental health information or asked about their exposure histories? *If yes*, what did you discuss? Did you ever do this before report-back?

15. Would you participate in a future clinic-based study that asked you to share results with participants? Why or why not?

16. Have you helped your patients, or the broader community engage in any advocacy, public education, or political activity?
    a. *If yes*, what did you do?
    b. *If not*, would you be interested in doing so? Why or why not?
    c. Do you feel prepared to act as a health advocate? Why or why not?

d. Did report-back change your perspective on engaging in environmental health policy?

IV. Final Thoughts

Thank you, I just have a few final questions for you.

17. Are you familiar with any additional resources that are helpful for clinicians who want to learn more about environmental hazards? *If yes,* which ones?

18. Is there anything else on which you would like to comment?

Thank you very much for your time and for talking with me so thoughtfully. Your input will be essential for the future design of how best to share chemical exposure results with study participants in clinical settings.
